# Spinal Exostosis in a Boy with Multiple Hereditary Exostoses

**DOI:** 10.1155/2013/758168

**Published:** 2013-11-10

**Authors:** Ali Al Kaissi, Rudolf Ganger, Klaus Klaushofer, Franz Grill

**Affiliations:** ^1^Ludwig Boltzmann Institute of Osteology, Hanusch Hospital of WGKK and AUVA Trauma Centre Meidling, First Medical Department Heinrich Collin Strasse 30 A, 1140 Vienna, Austria; ^2^Paediatric Department, Orthopädisches Spital Speising, Speisinger Strasse 109 A, 1130 Vienna, Austria

## Abstract

We report on a 13-year-old boy who presented with multiple hereditary exostosis and had development of back pain, associated with neurological deficits, and was found to have exostoses in the spinal canal. Spine radiograph showed a cauliflower-like abnormality of multiple exostoses of the posterior arch (pedicle) of the thoracic vertebrae (T3–5). Reformatted CT scanning revealed the simultaneous development of intra- and extraspinal osteochondromatosis of T3–5. The spinal cord was compressed by the intraspinal exostosis. Our patient was surgically treated for intraspinal exostoses and showed cessation of neurological deficits. We report what might be a rare association of spinal cord compression in a patient with multiple hereditary exostoses.

## 1. Introduction

The terms osteochondroma, osteocartilaginous exostosis, and exostosis are used interchangeably. Osteochondroma has been considered as the most common benign tumour of bone. These lesions constitute 10–15% of all bone tumours and 20–50% of all benign bone tumours. Although the pathogenesis of this lesion is not known, an abnormality or injury to the periphery of the growth plate has been suggested as the cause [1, 2]. The patient with a solitary exostosis is usually brought in by a parent who has noticed a mass adjacent to a joint. The patient usually experiences no symptoms. An occasional patient has loss motion in the adjacent to a joint, and this is attributable to the size of the mass. Exostoses are so characteristic on a plain radiograph that they can be diagnosed from their radiographic appearance alone. The mass is a combination of a radiolucent cartilaginous cap with varying amounts of ossification and calcification. The amount of calcification and bone formation increases with age. The base may be broad (sessile exostosis) or narrow (pedunculated exostosis). In the paediatric age group, exostosis should be expected to grow. They may continue to grow well into the third decade of life. The growth rate is not steady, and occasionally a lesion grows more rapidly than expected. Removal of the lesion in a child is indicated only for those patients who have symptoms attributable to pressure on a neurovascular bundle or irritation of the underlying muscle. Removal of the lesion in a young child may result in damage to the growth plate and recurrence of the lesion. Degeneration of the lesion into a malignancy is extremely rare in children and uncommon in adults. Gross examination of an exostosis reveals a lesion that looks like a cauliflower. It has an irregular surface covered with cartilage. The cartilage is usually less than 1 cm thick, except in the young child, in which it may be 2 or 3 cm thick. Deep in the cartilaginous cap, there is variable amount of calcification, enchondral ossification, and normal bone with a cortex and cancellous marrow cavity [1–4].

## 2. Case Report

A 13-year-old boy with hereditary multiple exostosis and was a known client in our department because of multiple hereditary exostoses. He underwent a series of operations previously: excision of the lesion of left femur, excision of right ulna, excision of right ulna, and regenerate fracture (casting for 3 weeks). Range of motion (ROM) is as follows: elbow 0–5–140; wrist 60–0–30, final ulna 1.5 cm shortened with subsequent development of subluxated radial head, though there was good wrist position (centred). 

**Figure 1:**
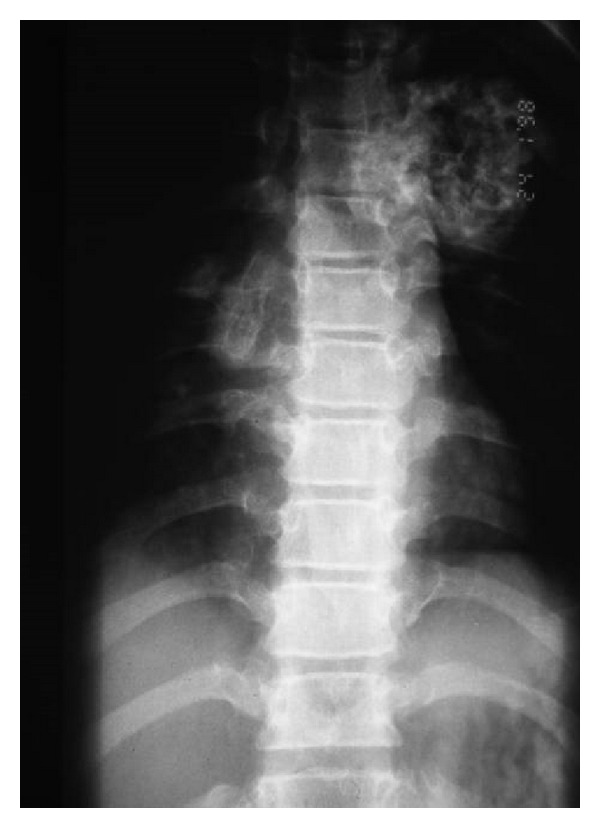
Conventional radiograph showed large cauliflower mass projecting along the anterolateral aspect of the spine.

**Figure 2:**
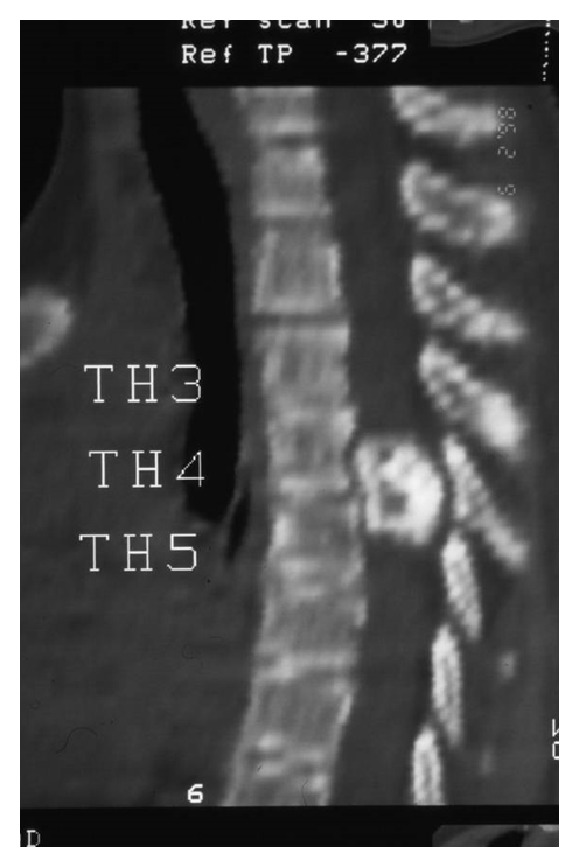
Reformatted sagittal CT scan showed a lesion arising from the T3–5 lamina and indenting the spinal cord (obliteration and stenosis of the spinal canal at the level of T3).

**Figure 3:**
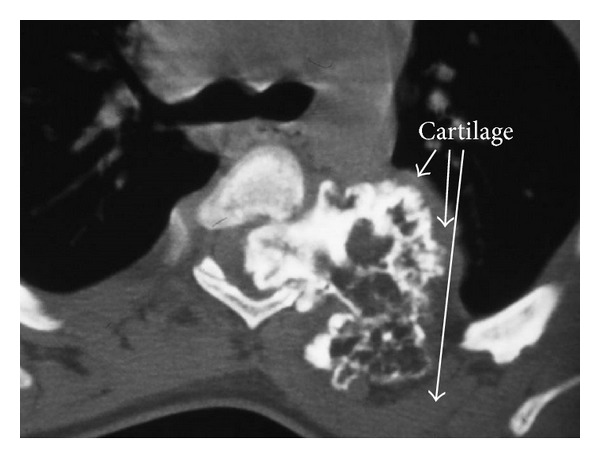
Axial CT of T3 scan showed the extent of the involvement of the left lamina and compression of the cord. The cauliflower appearance corresponds to the ossified matrix of the osteochondroma (huge intraspinal mass with heterogeneous density). This ossified matrix is surrounded by cartilage tissue, the osseous tumour originating from the left T3–5 facet joint (arrows).

**Figure 4:**
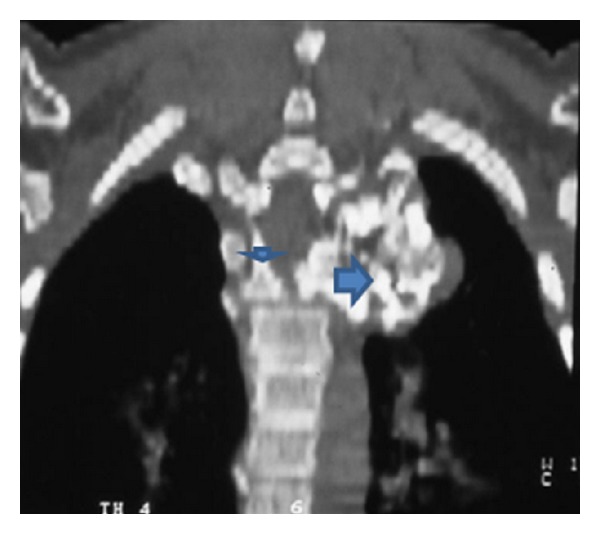
Coronal reformatted CT scan image showed a huge intraspinal mass with heterogeneous intensity. The ossified matrix is surrounded by cartilage tissue. The osseous tumour originating from the left T3–5 facet joint (arrows-arrow head is the pedicle and long arrow is the exostosis).

Recently, the patient developed pain and weakness in both lower limbs associated with urinary incontinence. Neurologic examination showed hyperreflexia, sustained clonus, Babniski sign, and decreased pinprick response to the level of the thigh in both legs. Conventional spine radiograph showed large cauliflower mass projecting along the anterolateral aspect of the spine (Figure 1). Reformatted sagittal CT scan showed a lesion arising from the T3–5 lamina and indenting the spinal cord (obliteration and stenosis of the spinal canal at the level of T3–5 (Figure 2). Axial CT of T3 scan showed the extent of the involvement of the left lamina and compression of the cord. The cauliflower appearance corresponds to the ossified matrix of the osteochondroma (huge intraspinal mass with heterogeneous density). This ossified matrix is surrounded by cartilage tissue, the osseous tumour originating from the left T3–5 facet joint (arrows) (Figure 3). Coronal reformatted CT scan image showed a huge intraspinal mass with heterogeneous intensity. The ossified matrix is surrounded by cartilage tissue. The osseous tumour originating from the left T3–5 facet joint (arrows-arrow head is the pedicle and long arrow is the exostosis) (Figure 4). Our patient underwent posterior decompression of T3–5. At operation an encapsulated mass was found arising from the posterior elements of T3–5 and causing compression of the spinal cord. Histological examination confirmed a benign osteochondroma. Typically, the microscopic appearance of the cartilaginous cap was that of benign hyaline cartilage. The patient made a remarkable neurological recovery in a period of 10 weeks. No symptomatic recurrence after the resection has been noticed.

## 3. Discussion

Multiple hereditary exostoses, also known as diaphyseal aclasis, are a common autosomal dominant inherited musculoskeletal disorder with a wide spectrum of clinical manifestations. It is characterized by the formation of multiple cartilage-capped exostoses arising from the region of the physis. The exact pathogenesis of the disorder is controversial and is not well understood despite much genetic and cellular molecular analysis, although the lesions are considered to be developmental hamartomas rather than true neoplasm. Osteochondromas are thought to arise in a peripheral portion of the growth plate. A focus of metaplastic cartilage forms and grows through progressive endochondral ossification, as a consequence of trauma or a congenital perichondral deficiency. Lesions may be radiation induced, in which case they are thought to be caused by a failure of the reserve cell layer in the epiphyseal growth zone. Radiation-induced osteochondromas constitute from 12–15% of lesions and occur more often when more than 25 Gy is given or when radiation is given to the very young (less than 2 years old) [1–4]. Previous reports described the potential for spinal cord compression in patients with multiple hereditary exostoses. Spinal osteochondromas are considered uncommon, reportedly accounting between 1–9% of all exostoses. This included all lesions of the spinal column, both within the spinal canal and those projecting away from the canal [5, 6]. Malignant degeneration into chondrosarcoma is rare, reported as 1–5% of solitary lesions. The risk of malignant degeneration is 10–25% in those with multiple hereditary exostoses [7].

 The cervical, thoracic, and lumbar region can be affected. Lower extremity discomfort associated with decreased balance, impaired coordination, spastic paraparesis, or other central neurologic dysfunction should raise the consideration of a vertebral osteochondroma. Between 1% and 4% of solitary osteochondromas arise in the spine, and 7–9% of patients with hereditary multiple exostoses develop a spinal lesion. Within the spine, lesions almost always occur in the posterior elements. Solitary lesions affect the cervical spine most commonly with a predilection for the atlantoaxial area, followed by the thoracic spine, then the lumbar region [8–11]. O'Brien et al. [12] emphasized that the intraspinal exostoses causing spinal cord compression must undergo surgical *excision*, as the recovery of neurological function after surgical treatment is excellent, and the recurrence rate is low, whereas asymptomatic extraspinal lesions may be treated by observation. Albrecht et al. [13] also reported good results with surgical resection, finding that 89% of symptomatic patients treated operatively reported improvement of symptoms. 

Solomon [14] reported an incidence of 9% of spinal osteochondromas in a series of 52 patients with hereditary multiple exostoses, all were asymptomatic. Compression of the spinal cord is an uncommon manifestation of osteochondroma. The neurological deficit is invariably the result of compression caused by an expanding lesion arising from the posterior elements. Less often, lesions causing neural compression originate from the vertebral bodies or heads of the rib. Both solitary and multiple osteochondromas affect males more frequently than females, and patients with multiple exostoses presenting with a spinal lesion are usually younger than those with a solitary osteochondroma. The neurological deficit is invariably the result of compression caused by an expanding lesion arising from the posterior elements. Less often, lesions causing neural compression originate from the vertebral bodies or heads of the rib.

## 4. In Summary

The prime clinical manifestation in patients with spinal exostosis is pain. Spinal cord compression, however, is a very rare entity in patients with multiple hereditary exostoses. Lesions are mostly originated from posterior vertebral elements and the incidence of spinal exostosis is 3–9% in patients with multiple hereditary exostosis. Finally, we wish to stress that CT scanning is the modality of choice in these patients.
